# Disruption of histamine/H_1_R signaling pathway represses cardiac differentiation and maturation of human induced pluripotent stem cells

**DOI:** 10.1186/s13287-020-1551-z

**Published:** 2020-03-04

**Authors:** Xiaowei Zhu, Suling Ding, Hui Li, Zhiwei Zhang, Lili Xu, Jian Wu, Xiangfei Wang, Yunzeng Zou, Xiangdong Yang, Junbo Ge

**Affiliations:** 1 Shanghai Institute of Cardiovascular Diseases, Zhongshan Hospital, Institutes of Biomedical Sciences, Fudan University, Shanghai, 200032 China; 2 Department of Cardiology, Zhongshan Hospital, Fudan University, Shanghai, 200032 China; 3 Institutes of Biomedical Sciences, Fudan University, Shanghai, 200032 China

**Keywords:** Human induced pluripotent stem cells, Cardiac differentiation, Histamine, Histamine receptor

## Abstract

**Background:**

The efficiency and quality of human induced pluripotent stem cell-derived cardiomyocytes (hiPSC-CMs) are crucial for regenerative medicine, disease modeling, drug screening, and the study of the development events during cardiac specification. However, their applications have been hampered by the differentiation efficiency, poor maturation, and high interline variability. Recent studies have reported that histamine plays important roles in hematopoietic stem cell proliferation and neutrophil maturation. However, its roles in cardiovascular tissue regeneration have not been thoroughly investigated. In the current study, we identified a novel physiological function of the histamine/histamine 1 receptor (H_1_R) signal in regulating the differentiation of hiPSC-CMs and heart development.

**Methods:**

Transgenic zebrafish model (cmlc2: mCherry) was treated with histamine and histamine receptor (HR) antagonists. Histological morphology and ultrastructure of zebrafish heart were measured. Histamine-deficient pregnant mice (HDC^−/−^) were treated with H_1_R antagonist (pyrilamine) by intragastric administration from E8.5 to E18.5. Cardiac histological morphology and ultrastructure were analyzed in neonatal mice, and cardiac function in adult mice was measured. In vitro, histamine and HR antagonists were administrated in the culture medium during hiPSC-CM differentiation at different stages. The efficiency and maturation of cardiac differentiation were evaluated. Finally, histamine-treated hiPSC-CMs were transplanted into ischemic myocardium to detect the possible therapeutic effect.

**Results:**

Administration of H_1_R antagonist during heart development induced cardiac dysplasia in zebrafish. Furthermore, using histidine decarboxylase (HDC) knockout mice, we examined abnormal swelling of myocardial mitochondria and autophagy formation under the condition of endogenous histamine deficiency. Histamine significantly promoted myocardial differentiation from human induced pluripotent stem cells (hiPSCs) with better structure and function via a H_1_R-dependent signal. The activation of histamine/H_1_R signaling pathway augmented hiPSC-derived cardiomyocyte (hiPSC-CM) differentiation through the ERK1/2-STAT3 signaling pathway. In addition, histamine-pre-treated hiPSC-CMs were transplanted into the ischemic hearts of myocardial injured mice and exhibited better survival and myocardial protection.

**Conclusions:**

Thus, these findings indicated that histamine/H_1_R and its downstream signals were not only involved in cardiac differentiation but also provided a better survival environment for stem cell transplanted into ischemic myocardium.

## Background

Ischemic heart disease caused by myocardial infarction (MI) is the leading cause of death worldwide [1, 2]. MI inflicts a permanent loss of cardiomyocytes (CMs), resulting in irreversible damage to cardiac function and ensuing by heart failure. Because of the limited regeneration capacity of adult CMs, the generation and maintenance of functional CMs are of critical importance for clinical applications. Numerous strategies have been rigorously explored to search for approaches to prevent or even reverse the pathological deterioration of heart tissue. Among these methods, cellular therapy using pluripotent stem cell-derived CMs, including both human embryonic stem cells (hESCs) and human induced pluripotent stem cells (hiPSCs), is propitious due to the similarities with human primary CMs. Despite substantial progress in the induction of cardiac differentiation, hiPSC-CMs exhibit developmental immaturity and low efficiency, which greatly limits their applications [3]. To fully realize the potential of hiPSCs, we need to understand more about the mechanism of cardiac differentiation and design strategies to improve the survival of hiPSC-CMs in the ischemic microenvironment.

Heart development is a multistep process involving mesoderm differentiation, cardiac progenitor cell formation, and cardiac maturation. The in vitro differentiation approaches of pluripotent stem cell-derived CMs mimic the cardiomyogenic lineage commitment in vivo. Thus, the approaches inducing cardiac differentiation mainly rely on the detailed mechanisms governing heart development [4]. Four major signaling pathways are involved in early cardiac differentiation, namely, BMP, TGFβ/activin/NODAL, Wnt, and FGF with highly specific temporal windows for effectiveness [5]. The most reproducible and efficient strategies involve stage-specific activation and inhibition of different signaling pathways in defined culture conditions, recapitulating key steps in cardiac development in the early embryos. Although the efficiency of cardiac differentiation has been upregulated over the past decades, it is still a key problem to obtain enough differentiated and mature CMs derived from hiPSCs. Numerous studies have focused on the underlying molecular mechanisms of cardiac differentiation: low oxygen environments, microRNAs, extracellular matrix, and paracrine factors [6–8]. Among these promoting methods, chemical molecules are especially potent due to their robust efficiency and economy. Additionally, using factors presented during postnatal heart development to promote cardiac differentiation and maturation is usually promising. For example, G-CSF, ascorbic acid, and thyroid hormone have been reported to promote the differentiation and maturation of hiPSC-CMs [7, 9, 10].

Histamine is a biogenic amine that has well-defined multiple roles in numerous biological processes [11, 12]. Histamine can be taken up in the diet, but endogenous histamine is generated through the conversion of l-histidine to histamine by the action of a unique enzyme, histidine decarboxylase (HDC) [13, 14]. The existence of histamine and histamine receptors in the heart has been known at least since the 1940s [15]; however, its roles in cardiovascular physiology are still not well understood. Histamine is of the neurohormonal factor that activates various cellular functions by stimulating histamine receptors [16]. Previous studies suggested that histamine H_2_ receptor antagonists are associated with heart failure protection [17]. However, our recent studies demonstrated that *HDC* knockout mice with histamine deficiency exhibited chronic inflammation abnormalities and aggravated cardiac injury [18, 19]. Recent studies report that HDC is expressed in hematopoietic stem cells and myeloid progenitor cells and that histamine deficiency in *HDC* knockout mice promotes myeloid cell proliferation [20]. Histamine has also been demonstrated to play pivotal roles in neurogenesis [21]. These data suggest that histamine/HR signaling plays a pivotal role in stem cell development and differentiation. However, the effects of histamine or HR antagonists on the development and differentiation of cardiac stem cells have been poorly studied.

In this study, using zebrafish models and histamine-deficient *HDC* knockout mice, we identified that the blockage of histamine/H_1_R signals using H_1_R antagonists could cause atrioventricular dysplasia during the early stage of cardiac myogenesis. Furthermore, an in vitro human induced pluripotent stem cell-based model was employed to explore the roles of histamine/HR signaling in cardiac differentiation. The results revealed that histamine/H_1_R signal, rather than H_2_R, could promote cardiac differentiation derived from hiPSCs via activating intracellular ERK1/2-STAT3 signaling. In support of our in vitro experiments, histamine-pre-treated hiPSC-CMs were transplanted into ischemic hearts of MI mice and exhibited better survival and myocardial protection. Therefore, our findings not only highlight a critical role of the histamine/H_1_R signaling pathway in cardiac myogenesis, but also provide a new strategy to improve the survival and efficiency of hiPSC-CMs after transplantation.

## Methods

### Treatment of zebrafish with histamine and histamine receptor antagonists

Wild type (laboratory AB-type) and transgenic (cmlc2: mcherry) provided by professor Yunzeng Zou from Fudan University were maintained on a 14-h light/10-h dark cycles. Embryos were obtained from natural spawning, raised at 28.5 °C in Embryo Medium (E3), and staged according to the standard protocol [22]. Phenylthiourea (PTU, 0.003%) was added to retard pigment formation until the desired stage. Ligand treatment of zebrafish embryo was done in 6-well plates. The experiments were divided into four groups: (a) control group, (b) histamine (1 mM) group, (c) pyrilamine (10 mM) group, and (d) cimetidine (100 mM) group. Thirty-five zebrafish embryos were kept in 3 ml E3 in each group. The treatment was started at 10 h post-fertilization (hpf). At 10 hpf, the fish were dechorionated to ease the penetration of ligand into embryo. The E3 and ligands were changed once every day to fresh solutions. The behavior was tracked from 10 hpf until 96 hpf by video, recording the heart beating rates and the gross morphology. H&E, TEM, and immunostaining of zebrafish embryo heart were carried out. Pyrilamine and cimetidine were purchased from Sigma.

### Animals

The Experimental Animal Ethics Committee of Fudan University approved the animal research protocol. All the animal procedures were performed in accordance with the Guiding Principles in the Use and Care of Animals (NIH Publication No. 85-23, revised 1996). Male, wild type (BALB/c background) and HDC knockout (HDC^−/−^ BALB/c background) mice were used in this study. HDC knockout (HDC^−/−^) mice were generally provided by Professor Timothy C. Wang from Columbia University. BALB/C mice were purchased from the Department of Laboratory Animal Science, Fudan University. Mice were housed in a temperature-controlled environment with 12 h/12 h light/dark cycles. BALB/c mice were divided into four groups: wild type group, HDC^−/−^ group, pyrilamine-treated WT group, and pyrilamine-treated HDC^**−/−**^ group. Pyrilamine (4 mg/kg/day) were provided for pregnant HDC^−/−^ and wild type BALB/C mice through intragastric administration from E8.5 to E18.5 every day, which is the specific stage for heart development. In cell transplantation experiments, BALB/C mice were randomly divided into four groups (*n* = 7): sham group, MI group, hiPSC-CM MI group, and histamine-treated hiPSC-CM MI group.

### hiPSCs derived cardiomyocyte generation and cell culture

The hiPSC (ATCC-DYR0100) cell lines were purchased from Shanghai Academy of Life Science and were routinely maintained in commercially available mTeSR1 medium (stem cell technologies) on Matrigel-coated plates (BD bioscience) according to manufacturer’s instruction. Differentiation into cardiac lineage was carried out with the protocol described by Lian et al. [23]. When the cell confluence arrived at ~ 90%, the medium was changed to RPMI/B27 without insulin with 6 μM GSK3-β inhibitor CHIR99021 and maintained for 48 h. Then, replace the CHIR-containing culture medium with RPMI/B27 without the insulin medium and culture 24 h. At day 3, change the medium to RPMI/B27 without insulin with 5 μM Wnt inhibitor IWR-1 and maintain for 48 h. At day 5, change the medium back to RPMI/B27 without insulin and leave alone for 48 h. Finally, replace the medium with RPMI/B27 with insulin at day 7 and replace medium every 3 days with the same medium. The spontaneous beating of cardiomyocytes should first be visible at approximately day 10. Histamine and HR antagonist were added into the culture medium at different time points of cardiac differentiation to examine the function of histamine and HR antagonist. There are at least three repeated experiments for every group.

### Teratoma formation from hiPSCs

To form teratomas, approximately 2 million undifferentiated hiPSCs were harvested, mixed with Matrigel, and injected subcutaneously to immunodeficient mice (NOD-SCID mice) according to procedure described by Nelakanti et al. [24]. After 6–8 weeks, teratomas were dissected, fixed with 10% formaldehyde in PBS, embedded in paraffin wax, and sectioned and stained with H&E.

### Clone formation assay

Cells were seeded into 6-well plates (200 C918 cells/well, 1000 RPE cells/well) and cultured for 3–4 days. Clones were visualized by crystal violet staining and counted.

### Immunofluorescence analysis histology and imaging

Standard protocols were used for immunostaining, histology, and imaging of stable transgenic fluorescence according to the standard procedure described by Bevan et al. [25].

### Transmission electron microscopy

Conventional electron microscopy was performed as described previously [26]. Human iPSC-CMs and zebrafish embryos at 96 hpf were fixed with 2.5% glutaraldehyde for 1 h at room temperature, followed with three washes with PBS (15 min each), and post-fixed with 1% osmium tetroxide for 2–3 h. The samples were then dehydrated in a graded series of ethanol concentrations and in propylene oxide for 10 min. Next samples were embedded, sectioned at about 70 nm thickness, and stained with lead citrate. Micrographs were captured using a PHILIPS CM-120 transmission electron microscope (PHILIPS, Holland).

### Infarcted size determination

Mice were euthanized with an overdose of sodium pentobarbital, and their hearts were then removed. Heart specimens were cleared of blood with chilled PBS and were fixed in formalin, embedded in paraffin, and cut into 5-μM-thick sections. Masson’ trichrome staining was performed to determine collagen deposition according to the procedures describing by Mao et al. [27]. To quantify fibrosis, images were analyzed by using the image J software.

### Immunoblot analysis

Immunoblot analyses were performed according to the protocol described as previously [28]. Briefly, the hiPSCs and differentiated CMs were seeded on Matrigel-coated slides and cultured for 2–3 days to allow tough attachment. Cells were fixed in 4% paraformaldehyde for 30 min and permeabilized in 0.5% tritonX-100 (Sigma) for 15 min at room temperature. The cells were washed two times with PBS and blocked in 5% BSA at room temperature for 1 h. Then, they were incubated with primary antibodies at 4 °C overnight and detected by Dylight 488 or Dylight 594 conjugated secondary antibodies (Jackson ImmunoResearch). Nuclei were stained with DAPI (Sigma). A Leica TCS sp2 microscope was used for slide observation and image capture.

### Echocardiographic assessment

Transthoracic echocardiographic analysis was performed on mice after the sham or MI surgery as described using Visual Sonic high-resolution micro-imaging system (Vevo770, Visual Sonic Inc., CA) equipped with a linear 30-MHz probe (RMV 707B). The mice were anesthetized with 1–2% isoflurane and placed on a heating pad to maintain the body temperature; the left ventricular dimensions were quantified by digitally recorded two-dimensional short-axis M-mode tracing as the level of papillary muscles. Left ventricular wall thickness and internal dimensions were measured. LV ejection (LVEF) and LV fractional shorting (LVFS) were calculated by Vevo770 version 3.0.0 analysis software (Visual Sonic Inc., CA).

### Quantitative reverse transcription-PCR

Total RNA was isolated from various treatment groups of cells using TRIzol reagent (Invitrogen). cDNA was prepared using the RT Kit (Takara). Each PCR was performed with specific primers, and the SYBR Premix EX-Taq (Takara) was used to detect the gene expression by an applied Biosystems AB 7500 Real Time PCR system. The mRNA level was standardized to internal control (GAPDH) and expressed as fold changes. The nucleotide primer sequences were found in Additional file 7: Table S2.

### Flow cytometry analysis

The cell samples were dissociated as previously reported [29]. The hiPSC-CMs were digested with collagenase I (1 mg/ml Sigma) with DNase I (40 Unit/ml Sigma) in PBS for 20 min, followed by 0.025% trypsin/EDTA treatment for 5 min at 37 °C. For analysis of cTnT- and Nkx2.5-positive cardiomyocytes from differentiated hiPSCs, cells were fixed and permeabilized with fixation a permeabilization solution for 30 min (BD bioscience) and stained with the primary anti-cardiac tropoinT antibodies or isotype control antibodies for 1 h, washed and stained with appropriate secondary antibodies. After staining, the cells were washed, resuspended, and evaluated with FACSalibur (BD Biosciences) and CELLQuest software.

### Acute MI model and cell transplantation

Ligation of left anterior descending (LAD) coronary was performed as reported [18]. Briefly, BALB/C male mice were anesthetized by inhalation of isoflurane, were intubated with a 22-G intravenous catheter, and then were fully anesthetized with 1–2% isoflurane gas while being mechanically ventilated on a positive pressure ventilator. Hearts were exposed by left-sided minimal thoracotomy, and LAD coronary artery was ligated using 8-0 silk ligature that was placed around it. The chest cavity was closed. Sham-operated mice underwent the same surgical procedures except that the suture placed under the left anterior descending artery was not tied. The histamine- or PBS-treated cells during cardiac differentiation at day 3–5 were labeled with a red dye (Dil, excitation/emission = 644/665 nm) purchased from Sigma–Aldrich (St. Louis, MO). Approximately 30 μl of cell suspension (2 × 10^5^ cells) containing derived CMs with histamine or without histamine was injected into the border zone of the infarcted heart, and each mouse was injected with three loci. To minimize immune rejection, Cyspin was provided after cell injection to the MI mice. Two weeks later, the hearts were excised and analyzed by immunocytochemistry staining after echocardiography.

### Western blot analysis

Cells were homogenized in ice cold lysis buffer containing proteinase and phosphatase inhibitor cocktail. The primary antibodies utilized in this study were as follows: OCT4 (sc-5279; Santa Cruz Biotechnology), Nanog (A3233 ABclonal), NKX2.5 (ab91196; Abcam), α-actinin (6487; Cell Signaling Technology), CX43 (3512 Cell Signaling Technology), GATA4 (5851; Cell Signaling Technology), MEF2C (5030; Cell Signaling Technology), cTnI (ab47003; Abcam), p-ERK (4370; Cell Signaling Technology), ERK (5013 Cell Signaling Technology), β-Catenin (8480; Cell Signaling Technology), p-cadherin (2189; Cell Signaling Technology), p-STAT3 (RT1490; HuaBio), STAT3 (ET1605; HuaBio), and GAPDH (5174; Cell Signaling Technology). Blots were developed using an enhanced chemiluminescence reagent (Thermo Fisher Scientific, Waltham, MA, USA).

### Statistical analysis

Data are expressed as mean ± SEM of at least three independent experiments for each cellular experimental group and at least five independent experiments for each animal group. We evaluate the data with Student’s test, and we used a one-way analysis of variance for multiple comparisons. A value of *p* < 0.05 was considered significant.

## Results

### Histamine 1 receptor is essential to heart development in zebrafish and mice

**Fig. 1:**
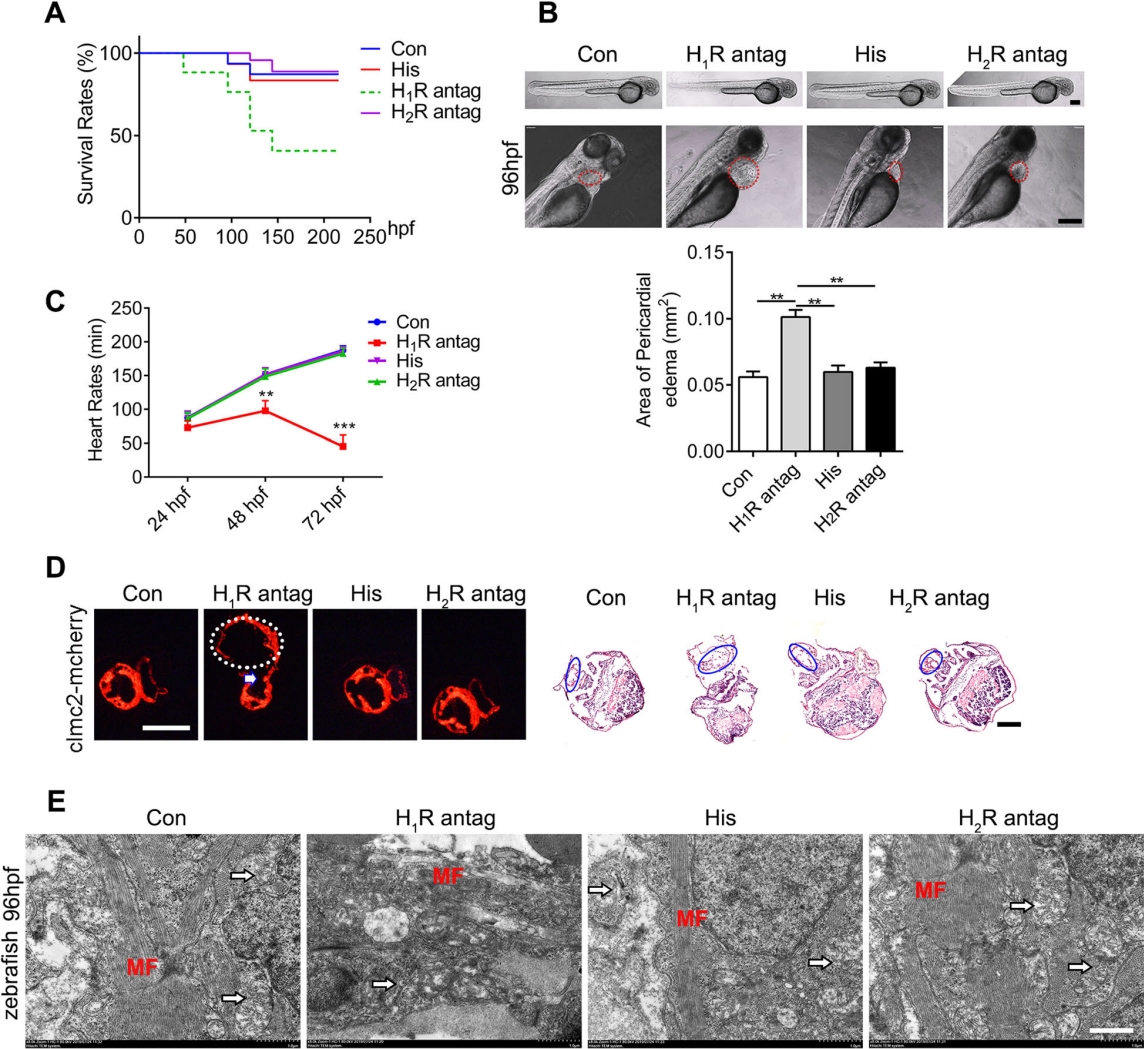
Histamine 1 receptor is essential to heart development in zebrafish. **a** Kaplan-Meier survival analysis at different time points post-histamine (his), H_1_R antagonist (pyrilamine), and H_2_R antagonist (cimetidine) treatment. **b** Representative microscope images of the cardiac morphology in zebrafish embryos at 96 h post-fertilization (hpf). The red dotted circles show cardiac morphology, and the quantification of pericardial edema area is shown below (*n* = 15, respectively). Scale bar 200 μM. **c** The heart rates of zebrafish under histamine, pyrilamine, and cimetidine treatment at different time points (*n* = 15 in each group). **d** Representative cardiac images of cmlc2-mCherry transgenic zebrafish embryos. The white dotted circles show enlarged atrial, and the white arrow shows septal defects (the left panel). H&E staining images of zebrafish are shown in the right panel and the blue circles show heart morphology (*n* = 10). Scale bar 100 μM. **e** Representative transmission electron micrographs of zebrafish heart at 96 hpf after administration of histamine, pyrilamine, and cimetidine (*n* = 4). The white arrows show cardiac mitochondria, and MF represents myofibroblasts. Scale bar 1 μM. Data are expressed as the mean ± SEM. **p < 0.01, ***p < 0.001 vs control

In order to explore the roles of histamine in heart development, a transgenic zebrafish model (*cmlc2*: mCherry) in which cardiomyocytes are specifically labeled with red dye was used in this study. First, histamine and histamine receptor (H_1_R and H_2_R) antagonists were administered into the culture medium of zebrafish at the embryo stage. We noticed that whereas histamine or histamine 2 receptor antagonist-cimetidine executed no obvious effects on the overall survival rates, blockage of histamine 1 receptor (H_1_R) by pyrilamine significantly decreased the survival rates compared with those of the control groups (80% vs 45%; log-rank test *p* = 0.0002) (Fig. 1a). Morphologic examination revealed that pyrilamine treatment led to severe pericardial edema as evidenced by an enlarged pericardial area, while histamine or histamine 2 receptor antagonist-cimetidine exposure minimally affected cardiac morphology in zebrafish (Fig. 1b). Meanwhile, the heart rates decreased significantly, and the rhythms were abnormal in the pyrilamine-treated group (Fig. 1c and Additional file 3: Video 1). Histological sections and H&E staining further confirmed that blockage of H_1_R by pyrilamine resulted in an enlarged atrial and obvious atrioventricular septum defects, and hardly looped to form a right-sided ventricle and left-sided atrium (Fig. 1d and Additional file 3: Video 1). Then, to further examine the effect of the histamine/HR signal on the ultrastructure of the zebrafish heart, transmission electron microscope (TEM) analysis was performed after histamine, pyrilamine, and cimetidine treatment at 96 h post-fertilization. The images demonstrated that pyrilamine treatment ruined the integration of the mitochondrial ridge of the zebrafish heart (Fig. 1e). These results collectively presented a critical role for H_1_R signaling in zebrafish cardiovascular development.

Although no significant effect of histamine was observed in zebrafish cardiac development, as shown in Fig. 1, we did not know whether it was due to its protective roles during this process and therefore hard to notice under normal conditions. Next, we employed an endogenous histamine deficiency mouse model in which the histidine decarboxylase (*HDC*) gene, the key enzyme responsible for histamine production, was deleted to investigate whether histamine/H_1_R signaling was involved in mouse heart development. Wild type (WT) and *HDC*^−/−^ pregnant mice were treated with or without pyrilamine from E8.5 to E18.5 through intragastric administration. Although the number of dead newborn mice slightly increased in the pyrilamine group compared with that in the control in *HDC*^−/−^ mice, no significant difference in the overall birth rates (Additional file 6: Table S1) and no apparent abnormalities in cardiac structure (Additional file 1: Figure S1A) were detected in neonatal mice born from WT and *HDC*^−/−^ pregnant mice post-pyrilamine treatment. To determine whether pyrilamine administration into pregnant mice affected adult heart function and structure, neonatal mice were fed to 8-week-old mice under normal feeding conditions and then examined by echocardiography. The echocardiographic parameters showed that endogenous histamine deficiency or pyrilamine treatment in pregnancy caused a mild reduction in the LVEF value, but no significant difference was achieved in the offspring of *HDC*^−/−^ mice compared with those of WT mice under normal conditions (Additional file 1: Figure S1B). Furthermore, the cardiac ultrastructure analysis by TEM revealed that the autophagosome accumulation was aggravated in the offspring mice from the *HDC*^−/−^ group administered pyrilamine during pregnancy (Additional file 1: Figure S1C). Our previous findings had revealed that histamine/H_1_R signaling executed protective roles in cardiomyocyte survival and cardiac function after acute myocardial infarction [18, 19]. In this study, no significant effect of the histamine/H_1_R signal on mouse heart development was observed, possibly because a trace of histamine contained in the diet is sufficient for cardiac development or because the maternal-infant barrier prohibits the efficiency of pyrilamine or because the role of the histamine/H_1_R signal in cardiac development is poorly conserved between zebrafish and mice. To exclude these problems, we designed an experiment to test the effect of histamine/H_1_R signaling on cardiomyocyte differentiation from human induced pluripotent stem cells (hiPSCs).

### Histamine has no effect on the maintenance of hiPSCs but promotes the efficiency of cardiac differentiation

The effect of histamine on the pluripotency of the hiPSC line was speculated at first. Neither low nor high concentrations of histamine affected the expression levels of pluripotent markers, such as *NANOG* and *OCT4*, as indicated by RT-qPCR (Additional file 2: Figure S2A), western blotting (Additional file 2: Figure S2B), and immunofluorescence staining (Additional file 2: Figure S2C). Similarly, histamine showed no obvious influence on the proliferative activity (Additional file 2: Figure S2D), the colony density (Additional file 2: Figure S2E), and the formation of teratomas with three germ layers (Additional file 2: Figure S2F).

**Fig. 2:**
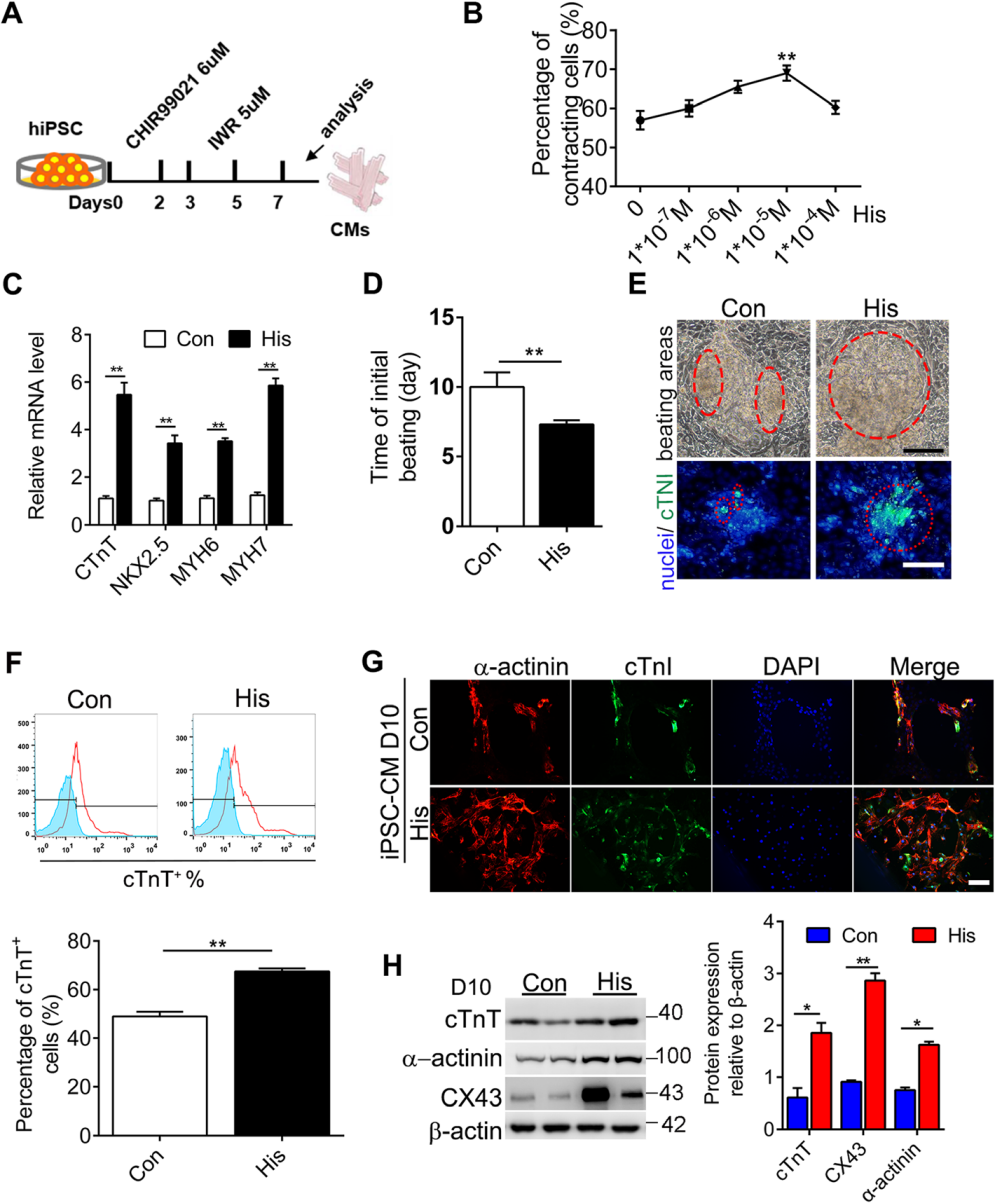
Histamine promotes the efficiency of cardiac differentiation. **a** Schematic timeline of the protocol used to direct human iPSCs into myocardial cells and the accompanying changes in cytokines. **b** The percentages of contracting CMs derived from hiPSCs under different concentrations of histamine treatment on day 10. **c** Gene expression of NKX2.5, cTnT, MYH6, and MYH7 in cardiomyocytes derived from hiPSCs under histamine (10^−5^ M) treatment. **d** The time required for the initial beating cardiomyocytes to appear. **e** Typical bright-field images of beating areas and immunofluorescence staining of cTNI in hiPSC-CMs on day 10. Scale bar 100 μM. The red dotted circles show the beating area. **f** Percentages of cTnT^+^ cardiomyocytes derived from hiPSCs on day 10 analyzed by FACS. **g** Immunofluorescence co-staining of α-actinin and cTnI (scale bar 100 μM) following histamine treatment. **h** Western blotting analysis of cTnT, α-actinin, and CX43 in hiPSC-CMs after histamine treatment. Data are expressed as the mean ± SEM. *p < 0.05, **p < 0.01, vs control (*n* = 3 for each group)

To test the effect of histamine on human cardiomyogenesis, we directed hiPSCs to undergo stepwise differentiation into cardiomyocytes according to a classical protocol [30]. Briefly, hiPSCs were treated with GSK3-β inhibitor CHIR99021 on days 1–2 to produce BRACHYURY^+^ primitive streak-like mesodermal progenitors followed by treatment with IWR-1 on days 3–5 to generate MESP1^+^ cardiogenic mesoderm cells. Usually, spontaneously beating cardiomyocytes initially appeared on days 8–10 (GiWi; Fig. 2a). To assess whether histamine impacted the process of hiPSC-derived myocardial differentiation, different concentrations of histamine were maintained for 7 days from the initiation of differentiation induction. The percentage of beating cells increased in a histamine dose-dependent manner and reached a peak at the concentration of 10 μM (Fig. 2b). RT-qPCR analysis showed that the levels of cardiac myofilament genes, including *cTnT*, *MYH6*, and *MYH7*, were dramatically upregulated under 10 μM histamine treatment (Fig. 2c). Moreover, 10 μM histamine treatment greatly shortened the time required for the initial beating cardiomyocytes to appear (Fig. 2d). Cell clone morphology and cTNI staining also exhibited obviously larger beating areas on day 10 after histamine treatment (Fig. 2e). Flow cytometry analysis revealed that the percentages of cTnT^**+**^ cardiomyocytes dramatically increased in the histamine-treated groups compared with the percentages in the control groups (Fig. 2f). The increased efficiency of myocardial differentiation induced by histamine was further confirmed by immunofluorescence co-staining of the cardiac-specific markers: α-actinin and cTnI (Fig. 2g). Consistently, the expression levels of myocardial markers including cTnT, α-actinin, and connexin 43 (CX43), were significantly increased in the histamine-treated group as shown by western blotting (Fig. 2h). To speculate about the carry-over effect of histamine treatment, the culture time was prolonged to 20 days. The histamine-treated group maintained a higher percentage of beating area and a higher beating frequency during the process (Additional file 2: Figure S2G). These data suggested that 10 μM histamine administration could enhance the efficiency of cardiac differentiation from hiPSCs.

### Histamine promotes the generation of cardiac progenitors from hiPSCs during directed cardiomyocyte differentiation

**Fig. 3:**
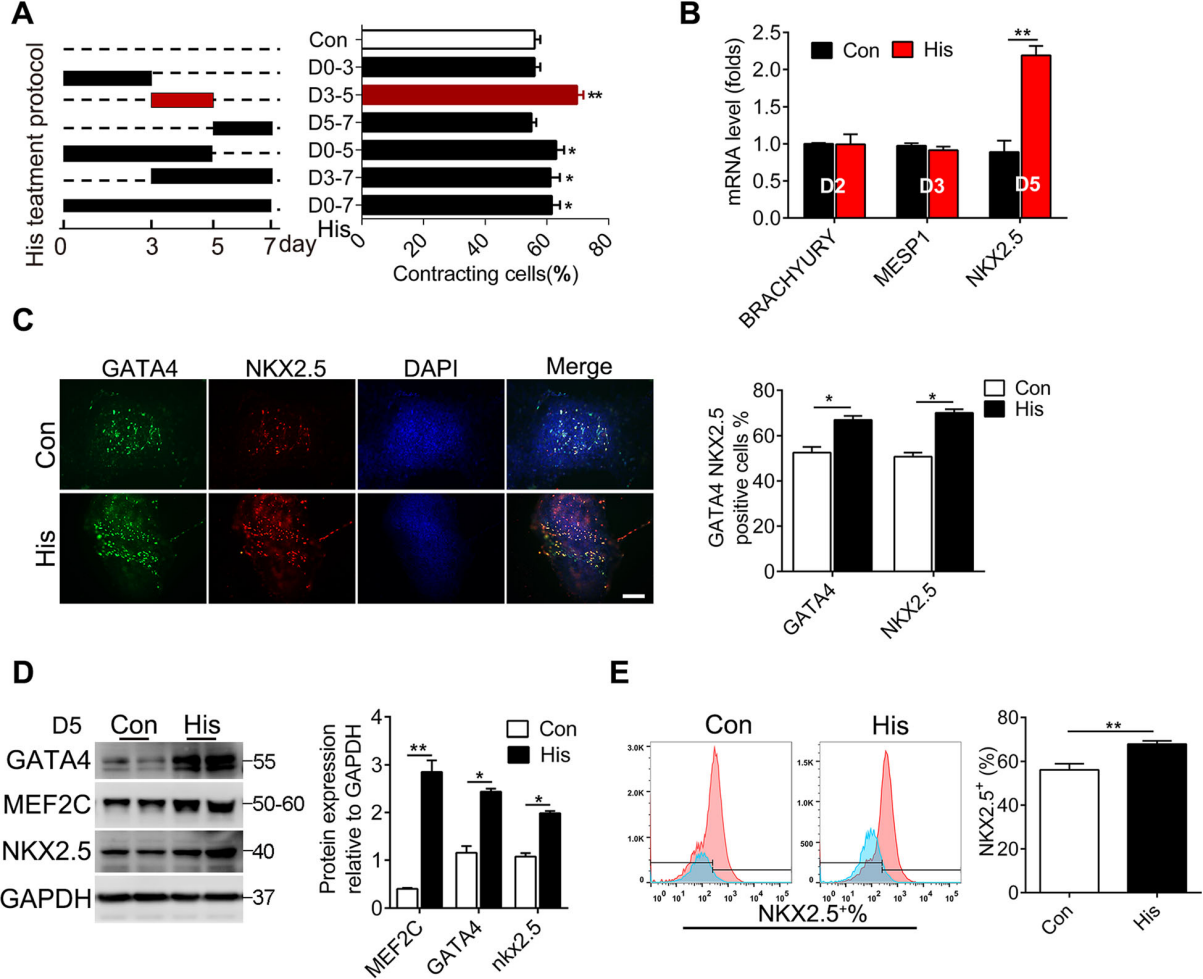
Histamine promotes the generation of cardiac progenitor cells from hiPSCs during directed cardiomyocyte differentiation. **a** Schematic diagram of the differentiation protocols (left panel); corresponding efficiency of cardiac differentiation of hiPSCs (right panel). **b** Gene expression of BRACHYURY, MESP1, and NKX2.5 respectively on days 2, 3, and 5 of differentiation. **c** Representative co-immunostaining views of GATA4 (green), NKX2.5 (red), and nuclei (blue) in cardiac progenitor cells on day 5 post-histamine administration. Scale bar 100 μM. **d** The protein levels of GATA4, MEF2C, and NKX2.5 in differentiated hiPSCs after 5 days of differentiation. The quantitative data are shown in the right panel. **e** The percentages of NKX2.5^+^ cells were analyzed by FACS. Data are expressed as mean ± SEM.*p < 0.05 vs control, **p < 0.01 vs control (*n* = 3 for each group)

The differentiation of cardiomyocytes from hiPSCs involves four stages including the primitive streak formation, the specification of the lateral mesoderm, and the generation of cardiac progenitors and cardiomyocytes. Next, to determine the critical stage at which histamine executed its function, 10 μM histamine was administered in several time-treatment patterns (Fig. 3a). The percentage of beating cells was measured on day 10, and the results showed that histamine treatment on days 0–3 failed to upregulate the percentages of beating cells. However, histamine exposure during days 3–5 or days 3–7 elevated the percentages of beating cells to a similar level as the 0–7-day histamine treatment pattern did (Fig. 3b), implying that histamine executed its function on advancing myocardial differentiation mainly at 3–5-day stage, which is the critical phase for the specification of the lateral mesoderm and the generation of cardiac progenitors. Histamine exposure during days 3 to 5 did not affect the marker expression of the lateral mesoderm (*MESP1* and *BRACHYURY*) but significantly augmented that of cardiac progenitor cells (*NKX2.5*) on day 5 (Fig. 3c), suggesting that the promoting effect of histamine was not on lateral mesoderm formation but on cardiac progenitor cell generation. The increased efficiency of cardiac progenitor cell generation in histamine-treated hiPSCs was further confirmed by immunofluorescence co-staining of the cardiac progenitor cell markers NKX2.5 and GATA4 (Fig. 3d), showing obviously more NKX2.5/GATA4 double positive cells in the histamine-treated derivatives. Moreover, the expression levels of cardiac progenitor cell genes, including GATA4, MEF2C, and NKX2.5, were significantly augmented in the histamine-treated group compared with that in the control group (Fig. 3e). The results of flow cytometry analysis confirmed that the percentages of NKX2.5^**+**^ cardiac progenitor cells were significantly increased in the histamine-treated group (Fig. 3f).

### Histamine facilitates the maturation of cardiomyocytes derived from hiPSCs

**Fig. 4:**
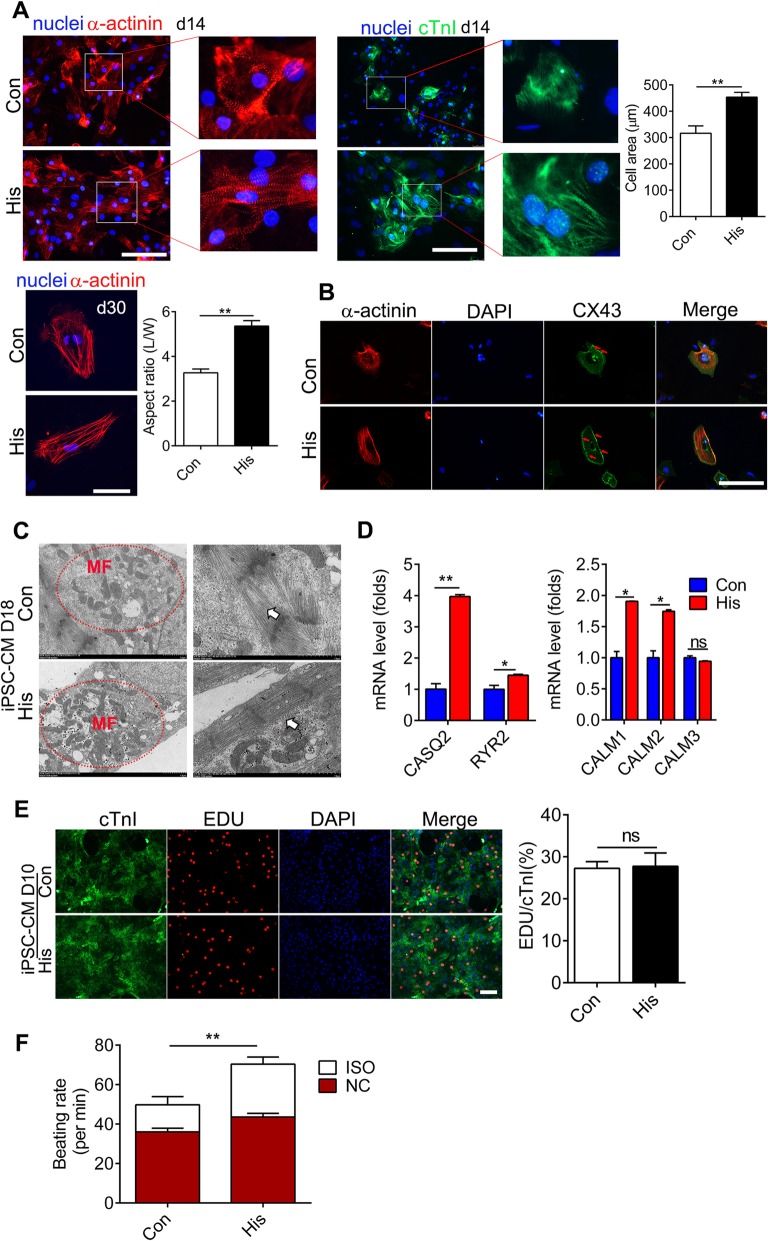
Histamine facilitates the maturation of cardiomyocytes derived from hiPSCs. **a** Sarcomeric structure analysis of hiPSC-CMs treated with histamine by α-actinin (red) and cTnI (green) staining on day 14 and day 30. Cell area was quantified on day 14, and the length-width ratio (L:W) was quantified on day 30. Scale bar 100 μM. **b** Representative co-immunostaining images of α-actinin (red) and CX43 (green) in hiPSC-CMs on the 14th day of histamine treatment. Scale bar 100 μM. **c** Representative transmission electron microscopy images of hiPSC-CMs on day18;white arrows indicate myofilament, and red dotted circles show cardiac mitochondria. **d** Gene expression of CASQ2, RYR2, and CALM1~3 of hiPSC-CMs. **e** Co-immunostaining of cTnI and EDU in hiPSC-CMs on day 14. Scale bar 100 μM. Data were quantified from 6 to 8 random fields in 3 assays. **f** The beating rates of hiPSC-CMs under the treatment of ISO. Data are expressed as the mean ± SEM. *p < 0.05, **p < 0.01 vs control (*n* = 3 for con group; *n* = 4 for his group)

Compared with adult cardiomyocytes, hiPSC-CMs are developmentally immature, exhibiting smaller size, and less organized sarcomeres and expression of fetal cardiac myofilament isoforms [31, 32], which hinders the clinical and research applications of hiPSC-CMs. To assess whether histamine treatment affected the maturation of the differentiated hiPSC-CMs, we investigated the morphological and structural properties of hiPSC-CMs differentiated from histamine-treated or histamine-untreated cardiac progenitor cells. The results of immunofluorescence staining of the myocardial markers α-actinin and cTnI showed that the morphological divergence between histamine-treated and histamine-untreated hiPSC-CMs emerged even on day 14, with histamine-treated hiPSC-CMs exhibiting more clear myofibrils and a larger size. Rod-like hPSC-CMs from histamine-treated CPCs had nearly double the L:W aspect ratio (3:1 vs 6:1) of hiPSC-CMs from untreated CPCs on day 30 (Fig. 4a), which indicates a relatively more mature level. The protein level of gap connexins is positively correlated with the level of maturation of cardiomyocytes and is also a biomarker of maturation of cardiomyocytes [33, 34]. Immunostaining of anti-connexin43 (CX43) revealed a higher level of CX43 expression in histamine-treated hiPSC-CMs (Fig. 4b). Myofibrils and sarcomeres are mature indicators related to the contraction force of cardiomyocytes [35, 36]. Ultrastructural characterization by TEM demonstrated that histamine treatment showed better-organized cross-striated myofilaments and more mitochondria in hiPSC-CMs on day 18 compared with those in the control group (Fig. 4c). Calcium (Ca^2+^) modulators are also key markers of mature cardiomyocytes [37]. The expression levels of crucial Ca^2+^ handling genes including *CASQ2*, *RYR2*, *CALM1*, and *CALM2* were dramatically higher in histamine-treated hiPSC-CMs (Fig. 4d). The speculation of the contractility capacities of hiPSC-CMs uncovered that histamine-treated hiPSC-CMs displayed quicker contracting rates on day 14 (Additional file 4: Video 2), which was consistent with the above results. Because the proliferation capacity of hiPSC-CMs usually decreased with advanced maturation, we speculated that histamine may affect the proliferation capacity of hiPSC-CMs. There was no significant alteration in the proliferative capacity of hiPSC-CMs as measured by the EDU incorporation rate (Fig. 4e). The β-adrenergic signaling pathway is the most important lusitropic regulator in cardiomyocytes [38]. Our results showed that the β-adrenergic agonist isoproterenol (Iso) at 10 nmol/l could significantly increase the beating frequency of histamine-treated hiPSC-CMs on day 14 to a much higher level compared with that of the control group, which again confirmed that the histamine-treated hiPSC-CMs were more mature (Fig. 4f).

### The function of histamine in the cardiac differentiation is mediated by histamine 1 receptor

**Fig. 5:**
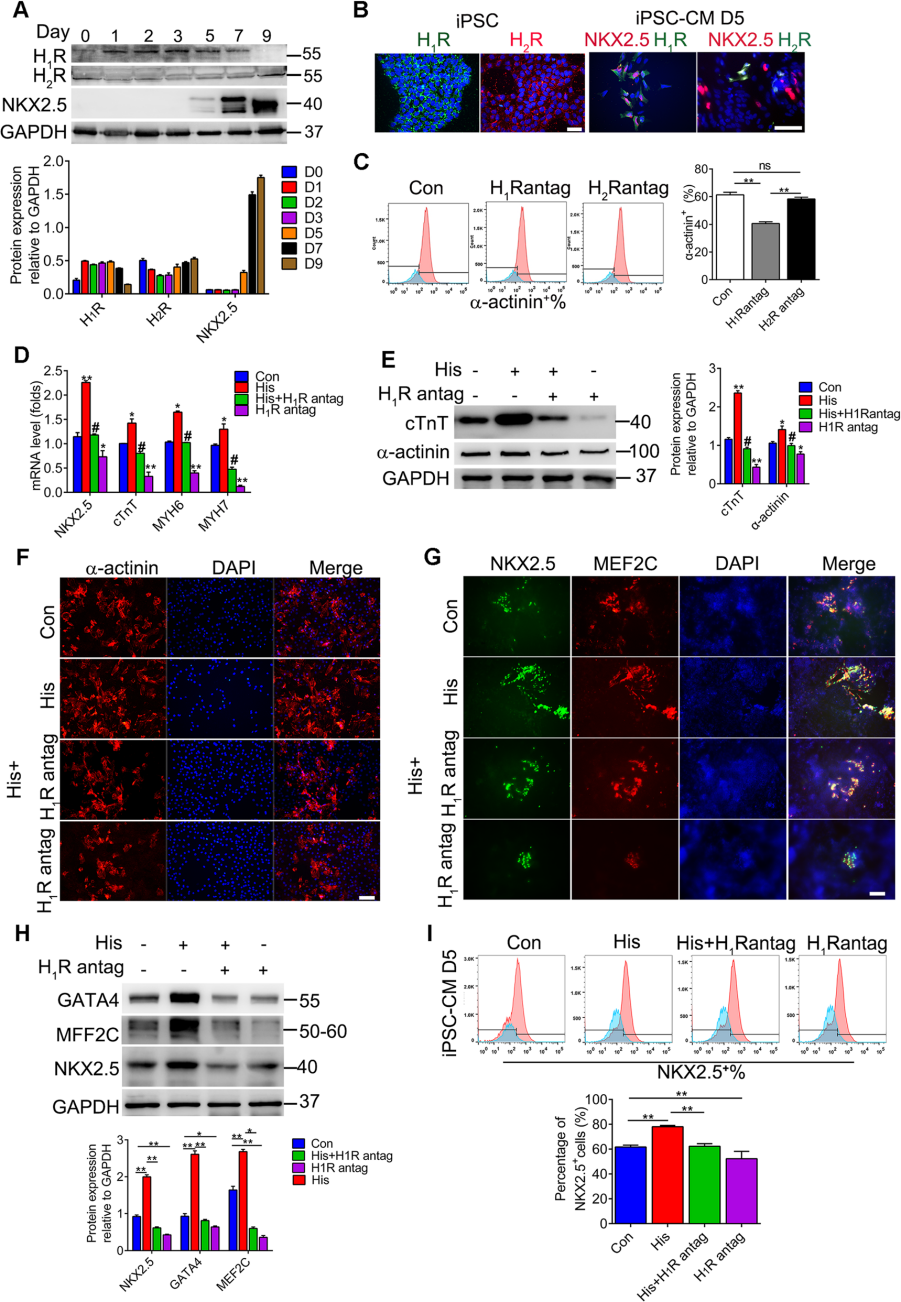
The function of histamine in the cardiac differentiation is mediated by the histamine 1 receptor. **a** The expression of H_1_R and H_2_R during cardiac differentiation was confirmed by immunoblot. Representative images and the densitometric analysis are shown. **b** Immunofluorescence staining for H_1_R, H_2_R, and NKX2.5 in hiPSCs and hiPSC-CPCs. Scale bar 100 μM. **c** Percentages of α-actinin^+^ cardiomyocytes on day 10 in the total population derived from iPSCs with or without pyrilamine and cimetidine treatment analyzed by FACS. **d** The mRNA levels of cardiogenic markers in hiPSC-CMs on day 10 with histamine and pyrilamine treatment. **e** The protein levels of α-actinin and cTnT in hiPSC-CMs on day 10 of differentiation. **f** Representative immunofluorescence images of а-actinin^+^ hiPSC-CMs. Scale bar 100 μM. **g** Representative immunostaining images of MEF2C (red), NKX2.5 (green), and nuclei (blue), in hiPSC-CPCs at day 5 of differentiation. Scale bar 200 μM. **h** The protein levels of GATA4, MEF2C, and NKX2.5 in hiPSC-CPCs on day 5. **i** Percentages of NKX2.5^+^ cells on day 5 in the total population derived from both hiPSC lines with or without histamine and pyrilamine treatment. Data are expressed as the mean ± SEM. *p < 0.05, **p < 0.01 vs control, ^#^*p* < 0.05 vs His (*n* = 3 for each group)

There are four known histamine receptors, H_1_R, H_2_R, H_3_R, and H_4_R, which are widely distributed in different tissues. To explore the underlying mechanisms of histamine in the process of cardiac differentiation, we determined the dynamic changes in the expression levels of four histamine receptors during cardiac differentiation. As shown in the above results, histamine exerted its influence on cardiac differentiation mainly from days 3 to 5. Although the qRT-PCR results showed that the mRNA levels of both *H*_*1*_*R* and *H*_*2*_*R* increased constantly during days 3 to 7 and reached a peak on day 7 (Additional file 5: Figure S3A), the western blotting results showed that only H_1_R possessed a high and stable protein level while the protein level of H_2_R was slight during days 3 to 5 (Fig. 5a). The protein level of H_3_R decreased during cardiac differentiation, while H_4_R was rarely expressed in iPSCs and cardiac cells (Additional file 5: Figure S3B). To further confirm the distribution of H_1_R and H_2_R, we performed immunostaining in hiPSCs and hiPSC-CMs. The results verified the equivalent expression of H_1_R and H_2_R in hiPSCs but much higher level of H_1_R in hiPSC-CPCs on day 5 (Fig. 5b). Next, we explored the possible receptors involved in cardiac differentiation by blocking H_1_R and H_2_R with the selective antagonists pyrilamine and cimetidine, respectively. Cell morphological observation showed that blockage of H_1_R by pyrilamine obviously affected the normal clone morphology, which was minimally affected by cimetidine compared with the control group at day 10 (Additional file 5: Figure S3C).

To confirm the essential roles of H_1_R in cardiac differentiation, we performed FACS analysis of cardiac cells on day 10. The results indicated that pyrilamine treatment not only suppressed the efficiency of cardiac differentiation but also abolished the pro-effect of histamine on cardiac differentiation (Fig. 5c), suggesting that H_1_R but not H_2_R was the mediator of histamine signaling in promoting cardiac differentiation. Moreover, the mRNA levels of the cardiac markers *NKX2.5*, *cTnT*, *MYH6*, and *MYH7* that were augmented by histamine were dramatically reduced by pyrilamine administration (Fig. 5d). Similarly, pyrilamine treatment repressed the protein levels of the cardiac makers cTnT and α-actinin increased by histamine (Fig. 5e). Immunostaining of α-actinin on day 10 revealed that pyrilamine obviously prevented the generation of α-actinin-positive cells promoted by histamine (Fig. 5f), confirming that the effect of histamine promoting cardiac differentiation was dependent on H_1_R. Next, we speculated whether H_1_R participated in the regulation by histamine of the generation of cardiac progenitors. Immunostaining of MEF2C and NKX2.5 on day 5 revealed that pyrilamine repressed the generation of cardiac progenitor cells promoted by histamine (Fig. 5g). The expression of GATA4, MEF2C, and NKX2.5 as evidenced by western blotting further proved the repressing effects of pyrilamine (Fig. 5h). The results of flow cytometry analysis confirmed that the increased percentages of NKX2.5^+^ cardiac progenitor cells in the histamine-treated groups were markedly reduced by pyrilamine treatment (Fig. 5i). In addition, pyrilamine treatment reversed the influence of histamine in shortening the time required for beating cells to appear, increasing the percentage of beating cells, and promoting the frequency of beating cells derived from hiPSCs (Additional file 5: Figure S3D). Collectively, these data demonstrated that H_1_R was the essential mediator of histamine in promoting cardiac progenitor cell generation and cardiac differentiation from hiPSCs.

### The ERK1/2-STAT3 pathway is the intracellular signal of histamine/H_1_R involved in enhancing cardiac differentiation

**Fig. 6:**
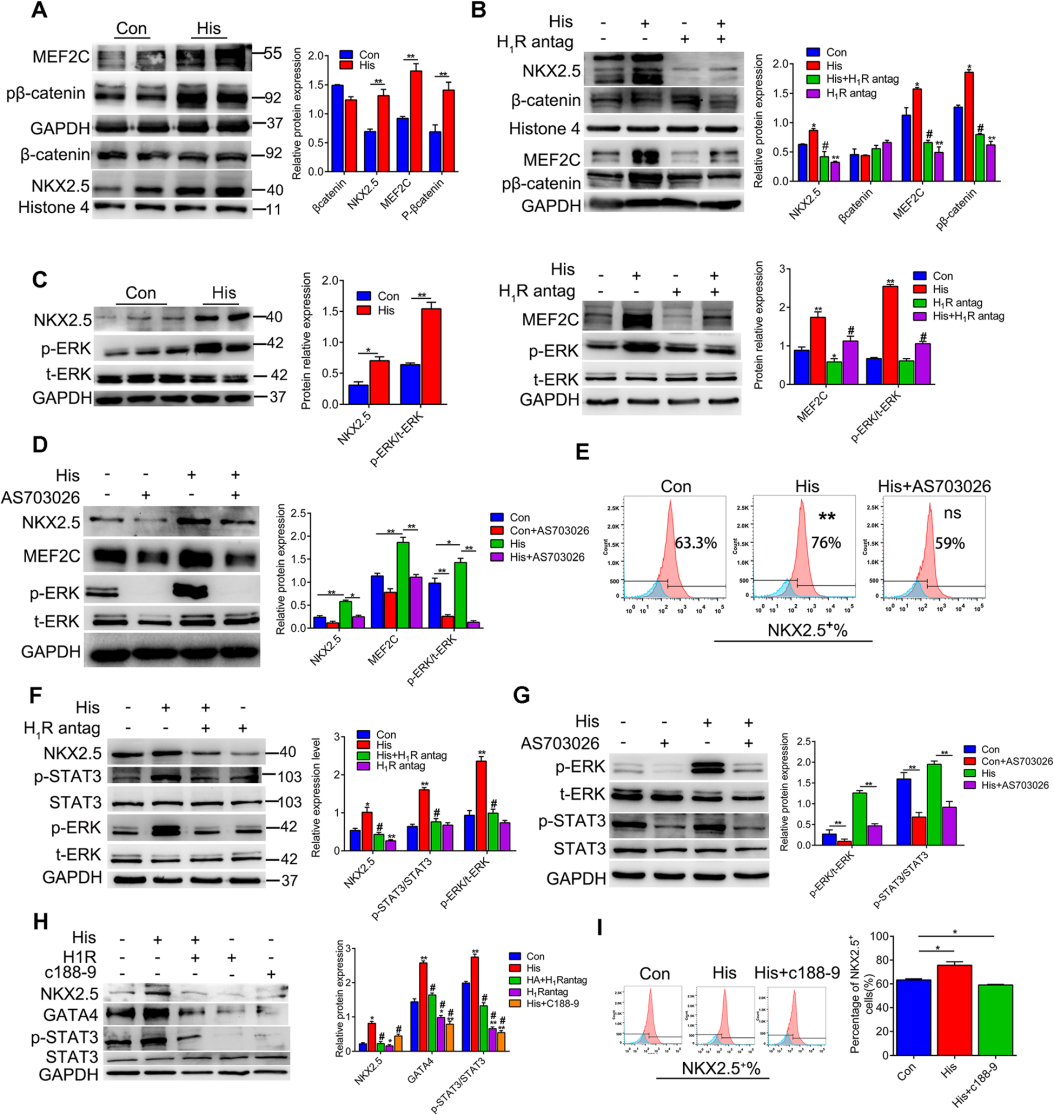
The ERK1/2-STAT3 pathway is the intracellular signal of histamine/H_1_R involved in enhancing cardiac differentiation. hiPSC were treated with histamine or pyrilamine on day 3 to 5 of cardiac differentiation. **a**–**c** Representative images of the protein levels of MEF2C, NKX2.5, phosphorylated β-catenin, β-catenin phosphorylated ERK, and total ERK along with the quantitative results. **d** hiPSC-CPCs were treated with or without the p-ERK inhibitor AS703026 on days 3–5 of differentiation. Representative immunoblots of NKX2.5, MEF2C, p-ERK, and t-ERK protein levels along with the quantitative results. **e** The percentages of NKX2.5^+^ cells under histamine and AS703026 treatment were analyzed by FACS. **f** The protein levels of MEF2C, NKX2.5, p-ERK, ERK, p-STAT3, and STAT3 along with the quantitative results. **g** Human iPSC-CPCs were treated with or without p-ERK inhibitor AS703026 on days 3–5 of differentiation. Representative immunoblots of p-ERK, t-ERK, p-STAT3, and STAT3 protein levels are shown along with the quantitative results. **h** The protein levels of cardiac genes (NKX2.5, GATA4) on day 5 post-treatment with histamine, pyrilamine, and p-STAT3 inhibitor C188-9 along with the quantitative results. **i** The percentages of NKX2.5-positive cells were identified by FACS. Representative univariate histograms and quantitative data are shown. Data are expressed as the mean ± SEM. *p < 0.05, **p < 0.01 vs control, ^#^*p* < 0.05 vs His (*n* = 3 for each group)

Next, we speculated that the intracellular pathway of histamine/H_1_R promotes cardiac differentiation. Given that the Wnt pathway was vital to the differentiation of cardiomyocytes, we explored whether histamine facilitated cardiac progenitor cell generation by affecting the expression of β-catenin. Histamine upregulated the level of phosphorylated β-catenin and increased the level of cardiac progenitor cell marker MEF2C (Fig. 6a). However, it was unexpected that histamine had no effect on the level of β-catenin in the nucleus (Fig. 6a). As expected, pyrilamine treatment abolished the consequence of histamine on the levels of the cardiac progenitor cell markers MEF2C and phosphorylated β-catenin (Fig. 6b). However, even pyrilamine treatment produced the reverse effect of histamine on the level of the cardiac progenitor cell marker NKX2.5 in the nucleus, and it did not affect the nuclear level of β-catenin (Fig. 6b). These results suggested that the effect of histamine/H_1_R on cardiac differentiation was probably not through the β-catenin signal.

Histamine has been widely reported to increase the intracellular Ca^2+^ influx [39], which would activate the downstream extracellular signal-regulated kinases 1 and 2 (ERK1/2) pathway [40]. The ERK1/2 signaling cascade is a key regulator involved in cell proliferation, differentiation, and survival [41]. The level of total ERK1/2 did not change, but the phosphorylation of ERK1/2 was intensified in response to histamine treatment accompanied by an increase of cardiac progenitor cell markers NKX2.5 (Fig. 6c). Blockage of H_1_R by pyrilamine reversed the effect of histamine on the level of p-ERK1/2 and the cardiac progenitor cell marker MEF2C (Fig. 6c). Moreover, when the phosphorylation of ERK1/2 was inhibited by the specific inhibitor AS703026, histamine exposure no longer increased the levels of the cardiac progenitor cell makers NKX2.5 and MEF2C (Fig. 6d). Flow cytometry analysis revealed that the increased percentage of NKX2.5^**+**^ cardiac progenitor cells in the histamine-treated groups was reduced by AS703026 (Fig. 6e), confirming that histamine/H_1_R signaling promoted cardiac progenitor cell generation by activating the ERK1/2 pathway.

Among the downstream factors regulated by the ERK1/2 pathway, we noticed that histamine treatment significantly promoted the tyrosine phosphorylation of STAT3 along with the increase of the cardiac progenitor cell markers NKX2.5 (Fig. 6f). Moreover, blockage of H_1_R by pyrilamine canceled the effect of histamine on the level of tyrosine phosphorylated STAT3 (Fig. 6f). Inhibiting the phosphorylation of ERK1/2 by AS703026 reduced the tyrosine phosphorylation of STAT3 that was augmented by histamine, as did pyrilamine (Fig. 6g). Similarly, the upregulation of the cardiac progenitor cell markers NKX2.5 and GATA4 induced by histamine was completely reduced by C188-9, the specific inhibitor of the tyrosine phosphorylation of STAT3, similar to when H_1_R was blocked by pyrilamine (Fig. 6h). The results of flow cytometry further verified that the histamine-increased percentages of NKX2.5^**+**^ cardiac progenitor cells were reduced by C188-9 as well by pyrilamine and AS703026 (Fig. 6i). Taken together, these results demonstrated that the function of histamine/H_1_R in promoting cardiac differentiation was dependent on the ERK1/2-STAT3 pathway.

### Histamine-preconditioned hiPSC-CMs improve cardiac functions after MI

**Fig. 7:**
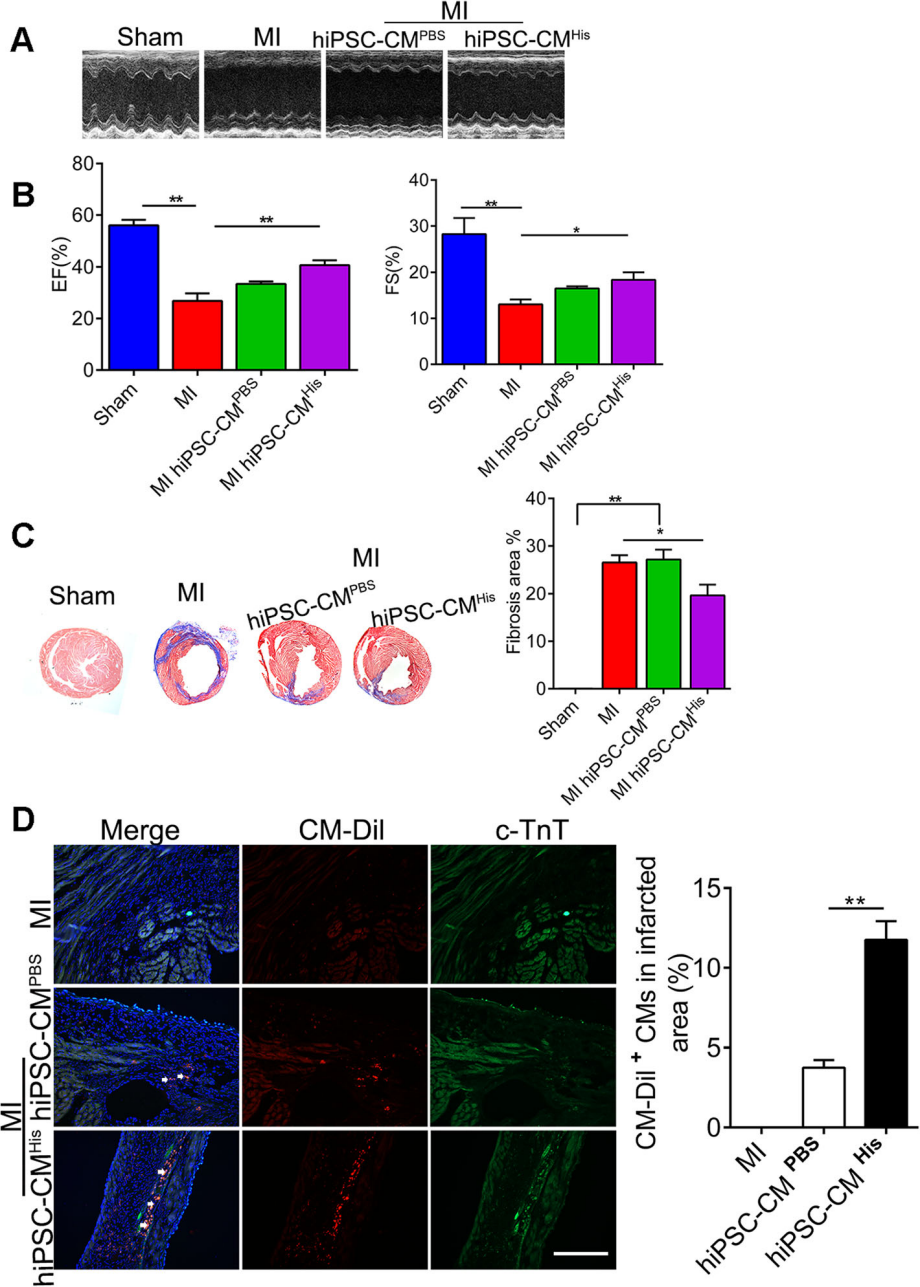
Histamine-preconditioned hiPSC-CMs improve cardiac function after MI. **a** Representative M-mode echocardiogram in sham and MI mice at 2 weeks after PBS- or histamine-treated hiPSC-CM implantation. **b** EF and FS of MI mice at 2 weeks after cell injection are shown. EF, ejection fraction; FS, fractional shortening. **c** Representative Masson’s trichrome staining of cardiac tissue obtained from mice administered with hiPSC-CMs. The quantitative results of infarcted size on day 14 post-MI or sham operation are shown. **d** Two weeks after cell implantation, the hiPSC-CMs were identified by CM-Dil (red) and cTnT, and the nucleus was stained with DAPI (blue) with the quantification of CM-Dil-positive cardiomyocytes in the injury area. Scale bar 100 μM. Data are expressed as the mean ± SEM. *p < 0.05, **p < 0.01 vs control (*n* = 7 for each group)

Human iPSC-derived cell therapy is becoming a promising strategy for the treatment of post-myocardial infarction (MI) intervention through complementing the lost cardiomyocytes. To evaluate the effects of histamine on the therapeutic potential of CMs, we labeled the control or histamine-preconditioned hiPSC-CMs with CM-Dil and transplanted them intramyocardially into the peri-infarct zone of the heart right after MI surgery. The formation of teratomas was not observed after transplantation of hiPSC-CMs. Cardiac function was assessed by echocardiography 2 weeks after transplantation. The results showed that hiPSC-CM transplantation improved heart function post-MI, but the histamine-preconditioned hiPSC-CM transplantation improved cardiac function to a more obvious degree than that of the PBS-treated hiPSC-CMs, as indicated by the augmentation of the ejection fraction (EF) and fractional shortening (FS) (Fig. 7a, b). Next, we performed Masson’s trichromatic staining to quantitatively analyze the infarcted size and the degree of fibrosis at 2 weeks post-MI. As indicated in Fig. 7c, both groups containing hiPSC-CMs showed a smaller infarct size and reduced fibrosis compared to the no hiPSC-CM group after MI. The transplantation of the histamine-preconditioned hiPSC-CMs reduced the infarct size and the fibrosis more markedly than did the PBS-treated hiPSC-CMs. The low retention rate and viability of transplanted cells in the insulted myocardium have hindered the further clinical transition of cell therapy [42]. In this study, microscopic images of the heart sections demonstrated that the survival/retention rate of hiPSC-CMs in the histamine-preconditioned group was significantly higher compared that of the PBS-preconditioned group, as indicated by the detection of CM-Dil-positive cells around the transplantation site at 14 days post-MI (Fig. 7d). Overall, this work corroborated a novel function of histamine in improving the cardiac therapeutic capacity of iPSCs, which has great potential in the treatment of MI.

## Discussion

Although much progress has been made in the field of cardiac differentiation, improving the differentiation efficiency and survival of transplanted cardiomyocytes is still a pressing problem. Comprehensively disclosing the factors and their mechanisms in cardiac differentiation is the crux of the matter. In this study, we found that the histamine/H_1_R axis was essential for cardiac development in zebrafish. Histamine/H_1_R signaling promoted cardiomyocyte differentiation and maturation derived from human iPSCs through activation of the ERK1/2-STAT3 pathway. Transplantation of histamine-treated hiPSC-CMs into injured hearts significantly improved cardiac function post-MI with higher cell retention and cell viability. These findings are helpful for obtaining a more comprehensive understanding of the mechanisms governing cardiac development. Furthermore, it provides a new opportunity for hiPSC-CM-based therapy.

Histamine, which is encoded by the HDC enzyme, has been demonstrated to be involved in cell proliferation, embryonic development, and tumor growth [12, 43–45]. However, its roles in cardiac differentiation and heart development have not been given enough attention over the past decades. This study revealed that histamine could significantly promote hiPSC differentiating toward cardiomyocytes for the first time. The generation of cardiomyocytes from PSCs was first reported using embryoid bodies with medium containing serum [46], but the efficiency was below 10%. Thereafter, various differentiation protocols involving growth factors and defined medium have been developed successfully to increase the cardiomyocyte differentiation efficiency. However, they have significant limitations, such as complex operation and high cost. Histamine is an endogenous active substance that is widely distributed in vivo. Many achievements have been made in the characterization of histamine and its receptors over the past decades. This undoubtedly would benefit the application of histamine or its downstream signal agonists as a safe and economical factor promoting cardiac differentiation for clinical and research use.

The distinct biological effects of histamine are mediated via the activation of its specific receptors, which differ in their tissue expression patterns and functions. A growing body of evidence has revealed that histamine can elicit a variety of responses in most parts of the cardiovascular system through its H_1_ and H_2_ receptors. In our study, it was observed that the H_1_ receptor possessed a stable and higher protein level at the initial stage of cardiac differentiation, and the promoting effect of histamine on cardiac differentiation derived from hiPSCs was dependent on the H_1_ receptor but not the H_2_ receptor. Because of the well-known functions of histamine in allergic and inflammatory reactions [47] and gastric acid secretion [12], anti-histamines (the specific antagonists of histamine receptors) are always prescribed to treat nausea and vomiting, which occurs in approximately 85% of pregnancies especially in the first trimester [48]. Given the wide usage of anti-histamines during the critical time for fetal development, there is a compelling need to examine any potential risks arising from their use. However, the necessary trials are ethically unacceptable [49], and the research on the safety of anti-histamines during pregnancy depends on the meta-analyses of all available observational cohorts. Unfortunately, the conclusions from these meta-analyses are still contradictory [50, 51], leaving questions unanswered. The important roles of histamine/H_1_R signaling in cardiac differentiation observed in this study remind us to pay more attention to the usage of H_1_R antagonists and the choice of anti-histamines during the early stage of pregnancy.

The in vitro cardiac differentiation from PSCs is similar to heart development involving the primitive streak formation, the specification of the lateral mesoderm, and the generation of cardiac progenitor cells and cardiac maturation. Cardiac progenitor cell formation mainly occurred during days 3 to 5 in the chemically defined methods [52]. In this study, through the administration of histamine in different time periods, we revealed that histamine enhanced cardiac differentiation mainly through promoting cardiac progenitor cell generation. The mammalian heart possesses a limited regenerative capacity, and loss of cardiomyocytes is the primary cause of myocardial infarction (MI). CPC-based therapy has been proposed as a promising strategy for the treatment of MI and adverse heart remodeling [53]. The identification of the effect of histamine on CPC generation and its mechanisms will undoubtedly promote the development of CPC-based therapy for adult heart repair. In the present work, ERK1/2-STAT3 was identified as the intracellular pathway through which histamine promotes CPC generation. ERK signaling activated by histamine and relevant receptors have been reported [40, 54]. Consistent with other studies, we found that histamine could elevate the expression of phosphorylated ERK and that inhibition of H_1_R could reverse this effect. STAT3 is a transcription factor that regulates the cellular responses to diverse cytokines and growth factors [55]. Multiple studies have shown that the activation of STAT3 promotes cardiomyocyte survival and is beneficial for the heart [56]. However, the function of STAT3 in CPC generation and cardiac differentiation was first characterized in this work. The important role of STAT3 in cardiac differentiation is also supported by a newly published study. It was discovered that deletion of STAT3 in ESCs would affect the differentiation of ESCs into the mesoderm and cardiac lineage [57]. The mechanisms through which STAT3 regulates CPC generation and cardiac differentiation need to be elucidated in detail in the future work.

CMs derived from hiPSCs generally exhibit immature characteristics similar to embryonic or fetal stage CMs and are functionally and structurally different from mature CMs, greatly limiting their clinical applications. To overcome this critical issue, several new approaches, such as co-culture with non-CMs, prolonged culture time, addition of miRNAs or hormones, mechanical or electrical stimulation, and 3D tissue engineering, have been proposed to generate more mature hiPSC-CMs [58, 59]. However, these strategies still have limitations such as scalability, clinical compatibility, and cellular damage which remained to be answered. More efforts are required to develop optimal methods to generate large-scale mature hiPSC-CMs. In addition to promoting CPC formation, the histamine/H_1_R signal well presented the ability to accelerate the maturation of hiPSC-CMs, as indicated by the morphological and structural properties. The proliferation capacity of cardiomyocytes derived from hiPSCs usually declines with maturation [33, 58]. There was no significant difference in the proliferation capacity observed between the histamine-treated group and the control group on the 14th day, which we thought might be because the time of differentiation was not long enough. The characteristics of long-time cultured histamine-treated hiPSC-CMs will be comprehensively elucidated in the future work. PSC-derived cardiomyocytes are composed of ventricular-, atrial-, and nodal-like cells. The myocardial subtype to which histamine-treated hiPSC-CMs belonged to is another issue that needs to be addressed in the future work. However, our findings provide a new opportunity to develop an efficient and productive approach for the generation of mature CMs.

Previous studies have reported abnormal development of epithelial cells in the gastric chief lineage and myeloid subsets of 8-week-old HDC^−/−^ mice, suggesting an important role for histamine HDC-expressing tissues [60]. In the present work, there was no significant defect observed in the parturition rate and the survival rate in HDC^**−/−**^ mice, which might be because a mild dose of histamine derived from the diet was enough for heart development. An H_1_R antagonist was administered into pregnant mice during E8.5 to E18.5, but no apparent flaw was observed in the cardiac structure and function of the progeny. We could not exclude the possibility that the maternal-infant barrier prohibited the efficacy of the H_1_R antagonist and concealed the effect. It is also possible that the function of the histamine/H_1_R axis was conserved between zebrafish and humans but not in mice. However, more autophagosome accumulation was observed in the cardiac tissue of adult HDC^**−/−**^ mice (8 weeks old), which is consistent with our previous work. The accumulation of autophagosomes does not affect cardiac function under normal conditions but makes the heart more sensitive to ischemic stress. In the future, experimental means of eliminating interference from histamine in the diet and maternal/infant barriers or H_1_R knockout mice will be needed to explore the real role of histamine/H_1_R in mouse heart development.

Stem cell therapy has obtained extensive consensus as an effective method for post-myocardial infarction (MI) intervention to repair the injured heart [61]. However, the low retention rates and viability of transplanted cells in the insulted myocardium have hindered the clinical translation of iPSC-based therapy. In our work, we observed that transplantation of hiPSC-CMs indeed improved the cardiac function post-MI. Moreover, histamine-treated hiPSC-CMs showed a better therapeutic efficiency. The results of CM-Dil-labeled cells suggested that histamine treatment not only promotes cardiac differentiation but also increases the retention and survival rates of hiPSC-CMs in the infracted heart. However, the mechanism is still unclear. In our previous work, it was observed that histamine could mitigate cardiomyocyte death by regulating autophagy [18]. Whether histamine treatment promoting the survival of hiPSC-CMs in the infracted tissue is relevant to autophagy will be investigated in the future work. Death of transplanted cells is the key issue slowing research progress in cell therapy for many kinds of diseases including diabetes, Parkinson’s disease, and muscular dystrophy [62]. The pro-survival effect of histamine on hiPSC-CMs may result in the illumination of a cell therapy for these diseases.

## Conclusions

**Fig. 8:**
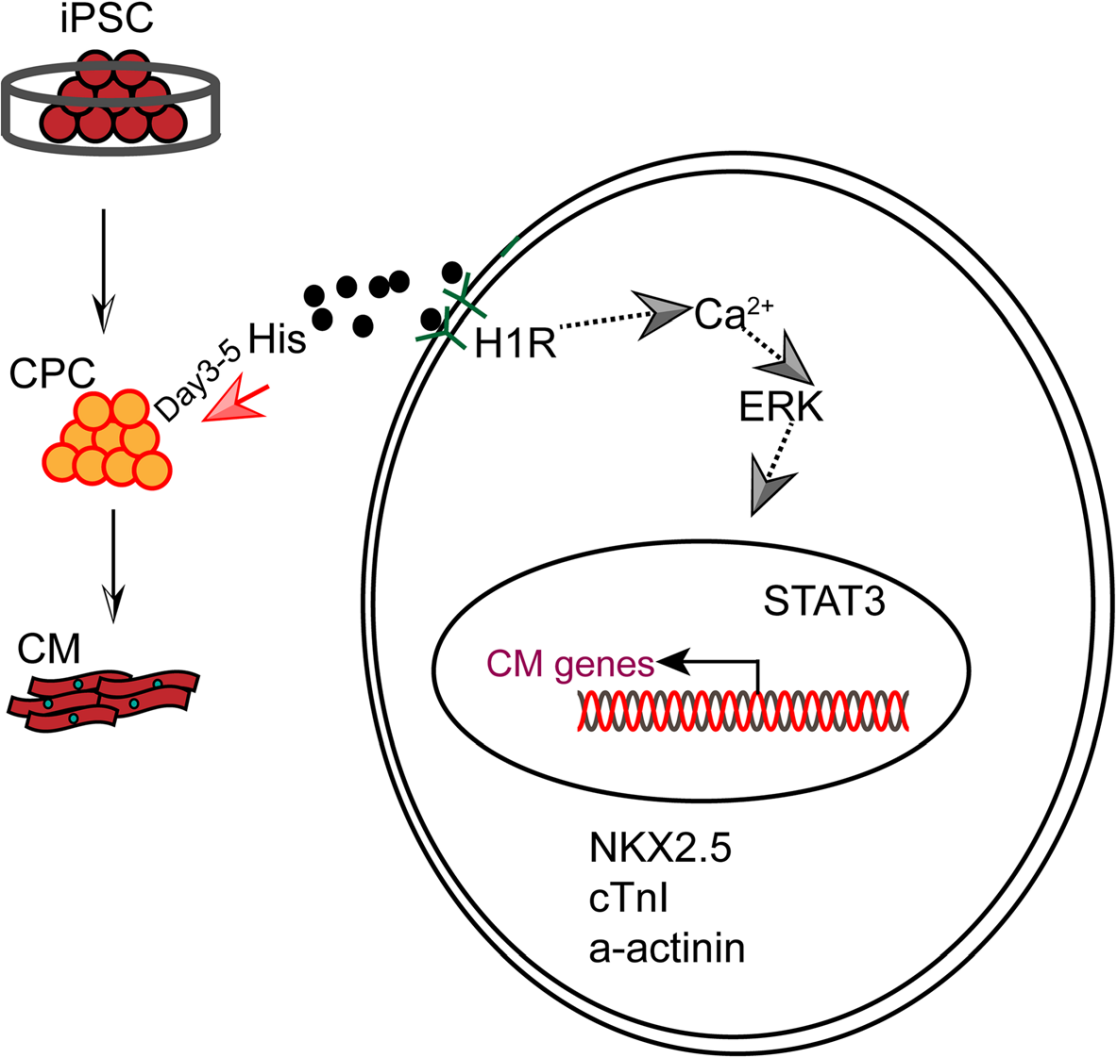
Schematic model of histamine/H_1_R signaling in regulating cardiomyocyte differentiation derived from human induced pluripotent stem cells

Our data have revealed a novel role of histamine/H_1_R in cardiomyocyte differentiation through activating the ERK1/2-STAT3 pathway (Fig. 8). These findings improve our understanding of the mechanisms of cardiomyocyte differentiation and cardiac development. Because of the multiple effects of histamine and its downstream signal agonists on CPC generation and cardiomyocyte maturation observed, we envisioned the great potential of histamine as a bioactive molecule to produce mature hiPSC-CMs for tissue engineering, drug screening, and regenerative medicine. Together, this work offers novel insights into the pathways driving cardiomyocyte generation and may offer new directions to improve cell-based therapies for cardiac diseases.

## Supplementary information

**Additional file 1 : Figure S1.** (related to Fig. 1). Inhibition of H_1_R minimally affected normal cardiac morphology and birth rates in mice. (A) Representative H&E staining sections of cardiac tissue obtained from neonatal WT and HDC^-/-^ mice on day 1 and day 7 after birth. The pregnant mice were treated with or without pyrilamine from E8.5 to E18.5 through intragastric administration (*n*=7, respectively). Scale bar 1mm. (B) Echocardiographic analysis of ejection fraction (EF), fractional shortening (FS), on day 28 after birth, mice were treated as A. Quantitative echocardiographic analysis is shown in the panel below. (*n*=6 for the control group and n=7 for each of the pyrilamine treated groups). (C) Representative cardiac electron micrographs in WT and HDC^-/-^ mice on day 28. Mice were treated as in A (*n*=4). The red dotted circles show the autophagosome, and the red arrow indicates the swelling mitochondria. Data are expressed as the mean ± SEM.

**Additional file 2 : Figure S2.** (related to Fig. 2). hiPSCs adapted to histamine retain stemness and full differentiation potential. (A) The mRNA levels of OCT4, Nanog, and SOX2 in histamine treated iPSCs at different concentrations. (B) Western blotting analysis of OCT4 and Nanog protein in hiPSCs post histamine treatment. (C) Immunofluorescence staining images of OCT4 in iPSCs. Scale bar 100 μM. (D) Presentative images of EDU staining in iPSCs. (E) The clone formation of histamine treated iPSCs at different concentrations. Scale bar 200 μM. (F) H&E staining of teratomas derived from iPSCs transplanted into immunodeficient mice. Scale bar 200 μM (G) Percentages of beating cells and frequency of beating of hiPSC-CMs after histamine treatment during days 3-5. Data are expressed as the mean ± SEM.*p < 0.5, ** *p* < 0.01 vs control.

**Additional file 3: : Video 1** The morphology and beating behavior of zebrafish after pyrilamine treatment at 96 hours post fertilization.

**Additional file 4: : Video 2** The morphology and beating rates of cardiomyocytes derived from hiPSCs after histamine treatment.

**Additional file 5 : Figure S3.** (related to Fig. 5). The function of histamine in cardiac differentiation may be mediated by histamine 1 receptor. (A) The mRNA levels of H1R, and H_2_R during cardiac differentiation. (B) The protein levels of H3R and H4R during cardiac differentiation. The quantified data are shown below. (C) Bright-field images of the typical morphology of hiPSC-CMs on day 7 post histamine, pyrilamine and cimetidine treatment during days 3 to 5. Scale bar 500 μM. (D) The initial beating time (right panel), frequency of contraction of hiPSC-CMs (middle panel), and percentage of beating cells on the indicated days (left panel). Cells were treated with pyrilamine and histamine on day 3th-5th. Data are expressed as the mean ± SEM. *p < 0.5, ** p < 0.01 vs control.

**Additional file 6 : Table S1.** Fertility and mortality in HDC^-/-^mice the overall birth and mortality rates of pregnant WT and HDC^-/-^ mice post pyrilamine intragastric administration during E8.5 to E18.5.

**Additional file 7 **: **Table S2.** Quantitative real time PCR primers.

## Data Availability

The data collected and the analysis performed to generate the manuscript results are available from the corresponding author on reasonable request. The datasets supporting the results of this article are included within the article.

## Abbreviations

hiPSCs: Human induced pluripotent stem cells
hiPSC-CMs: Human induced pluripotent stem cell-derived cardiomyocytes
CPCs: Cardiac progenitor cells
His: Histamine
HR: Histamine receptor
EF: Ejection fractional
FS: Fractional shortening
ERK: Extracellular signal-regulated kinase
STAT3: Transducer and activator of transcription 3
