# Supplementary figures and images for: Disruption of histamine/H1R signaling pathway represses cardiac differentiation and maturation of human induced pluripotent stem cells
Source: Stem Cell Res Ther. 2020 Mar 4;11:27. doi: 10.1186/s13287-020-1551-z (PMC7055148; doi:10.1186/s13287-020-1551-z)

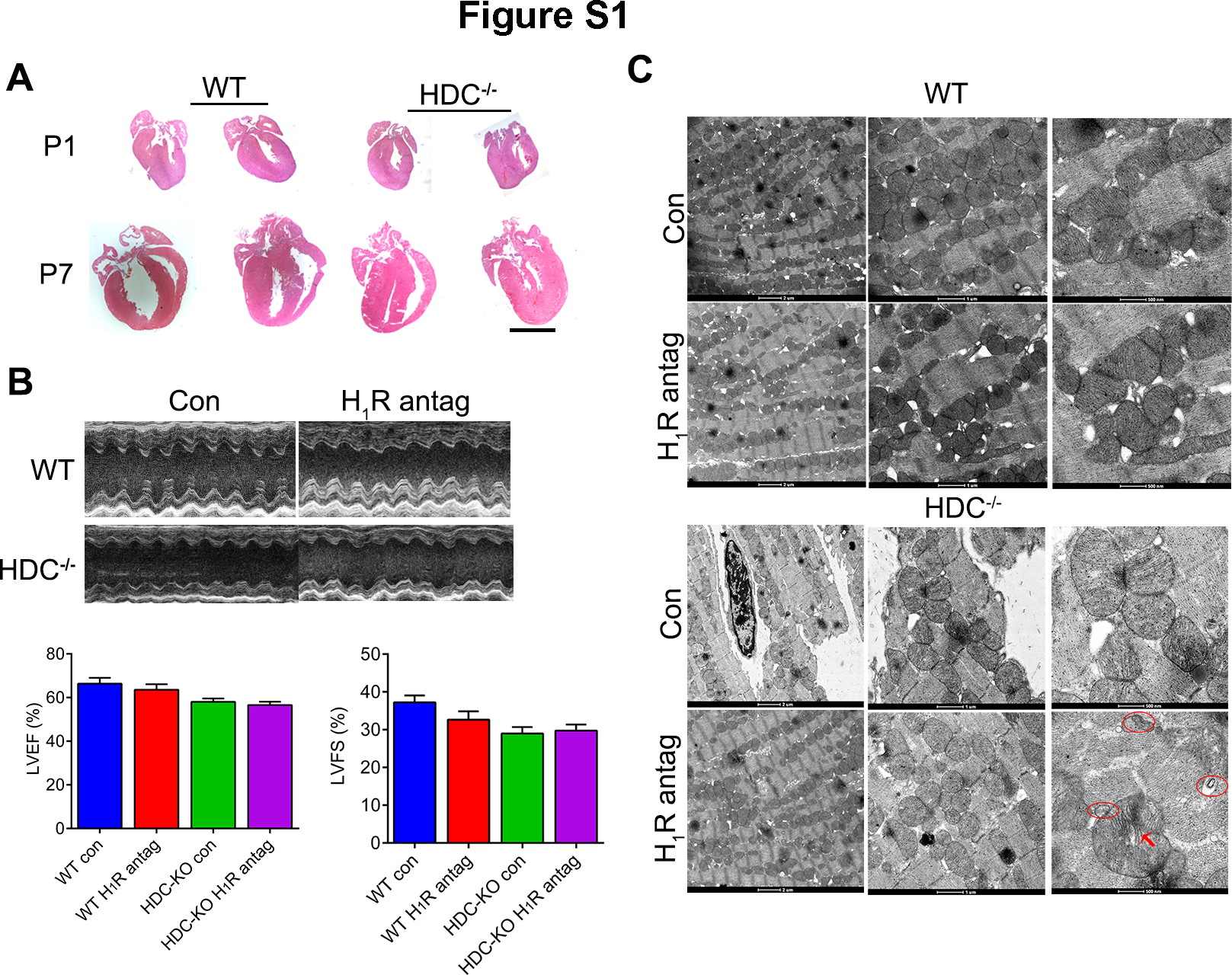

Supplement: Supplementary file 1 — Additional file 1 : Figure S1. (related to Fig. 1). Inhibition of H1R minimally affected normal cardiac morphology and birth rates in mice. (A) Representative H&E staining sections of cardiac tissue obtained from neonatal WT and HDC-/- mice on day 1 and day 7 after birth. The pregnant mice were treated with or without pyrilamine from E8.5 to E18.5 through intragastric administration (n=7, respectively). Scale bar 1mm. (B) Echocardiographic analysis of ejection fraction (EF), fractional shortening (FS), on day 28 after birth, mice were treated as A. Quantitative echocardiographic analysis is shown in the panel below. (n=6 for the control group and n=7 for each of the pyrilamine treated groups). (C) Representative cardiac electron micrographs in WT and HDC-/- mice on day 28. Mice were treated as in A (n=4). The red dotted circles show the autophagosome, and the red arrow indicates the swelling mitochondria. Data are expressed as the mean ± SEM. [file 13287_2020_1551_MOESM1_ESM.tif]

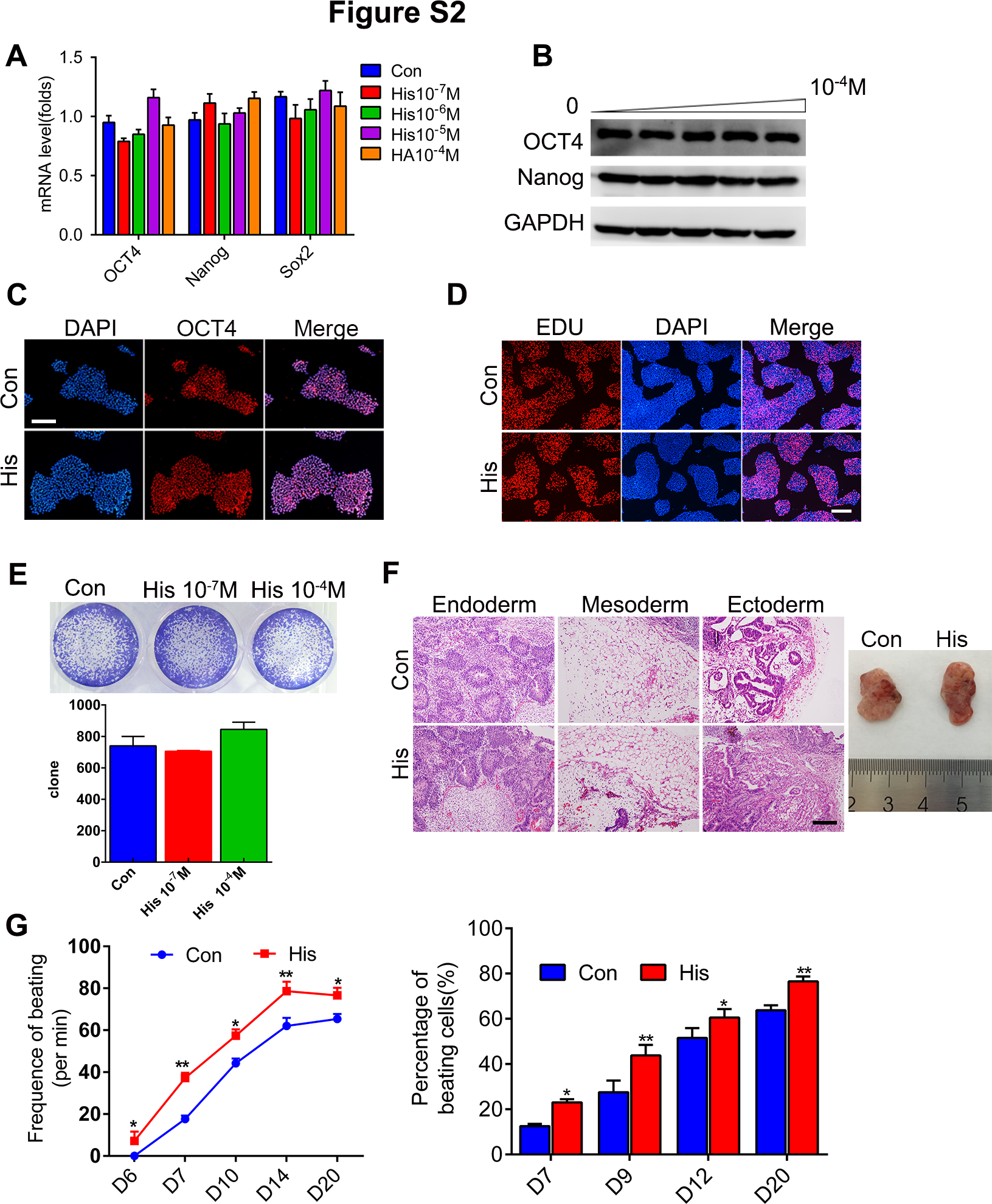

Supplement: Supplementary file 2 — Additional file 2 : Figure S2. (related to Fig. 2). hiPSCs adapted to histamine retain stemness and full differentiation potential. (A) The mRNA levels of OCT4, Nanog, and SOX2 in histamine treated iPSCs at different concentrations. (B) Western blotting analysis of OCT4 and Nanog protein in hiPSCs post histamine treatment. (C) Immunofluorescence staining images of OCT4 in iPSCs. Scale bar 100 μM. (D) Presentative images of EDU staining in iPSCs. (E) The clone formation of histamine treated iPSCs at different concentrations. Scale bar 200 μM. (F) H&E staining of teratomas derived from iPSCs transplanted into immunodeficient mice. Scale bar 200 μM (G) Percentages of beating cells and frequency of beating of hiPSC-CMs after histamine treatment during days 3-5. Data are expressed as the mean ± SEM.*p < 0.5, ** p < 0.01 vs control. [file 13287_2020_1551_MOESM2_ESM.tif]

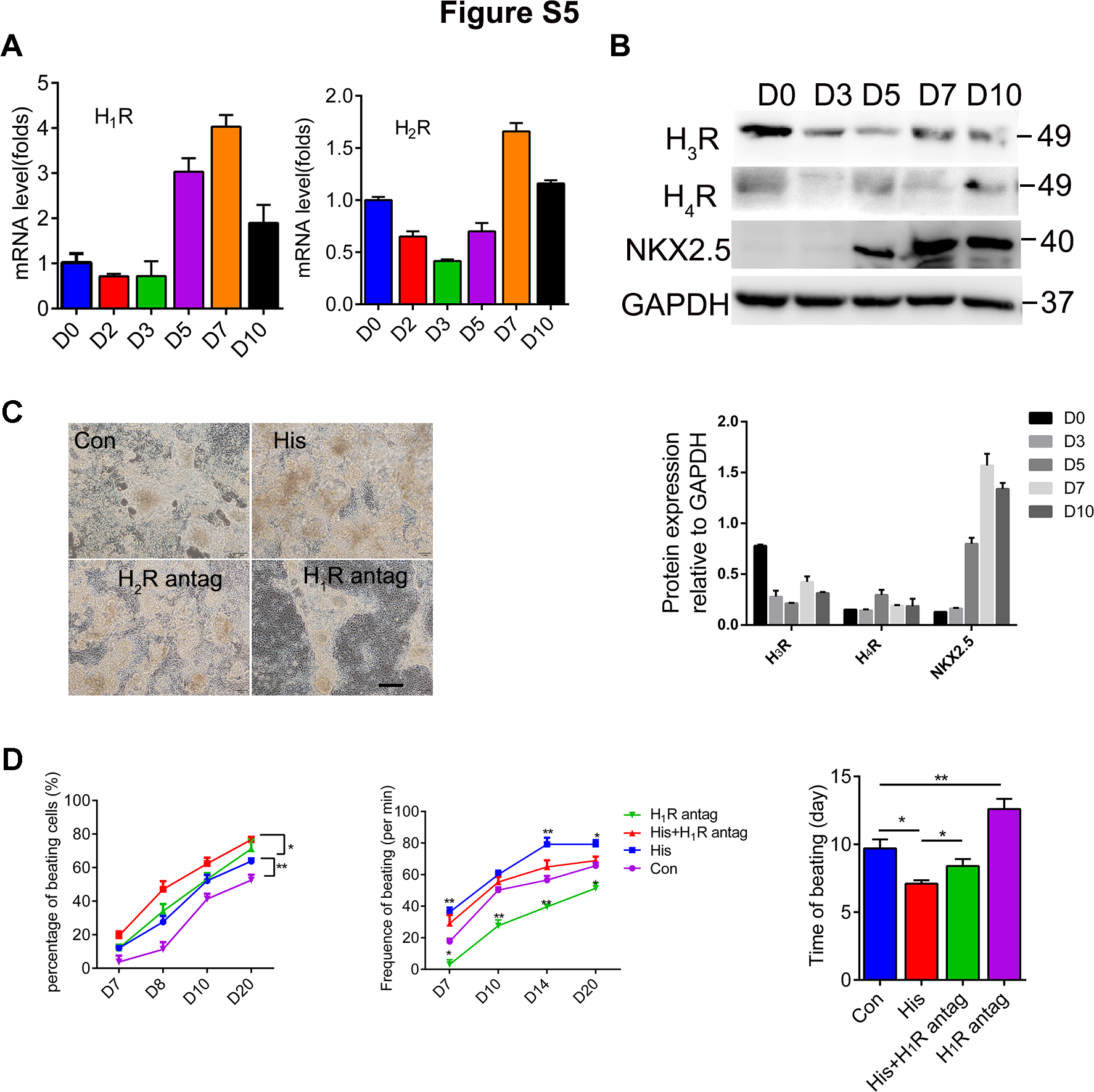

Supplement: Supplementary file 5 — Additional file 5 : Figure S3. (related to Fig. 5). The function of histamine in cardiac differentiation may be mediated by histamine 1 receptor. (A) The mRNA levels of H1R, and H2R during cardiac differentiation. (B) The protein levels of H3R and H4R during cardiac differentiation. The quantified data are shown below. (C) Bright-field images of the typical morphology of hiPSC-CMs on day 7 post histamine, pyrilamine and cimetidine treatment during days 3 to 5. Scale bar 500 μM. (D) The initial beating time (right panel), frequency of contraction of hiPSC-CMs (middle panel), and percentage of beating cells on the indicated days (left panel). Cells were treated with pyrilamine and histamine on day 3th-5th. Data are expressed as the mean ± SEM. *p < 0.5, ** p < 0.01 vs control. [file 13287_2020_1551_MOESM5_ESM.tif]
