# Supplementary material for: Disruption of histamine/H1R signaling pathway represses cardiac differentiation and maturation of human induced pluripotent stem cells
Source: Stem Cell Res Ther. 2020 Mar 4;11:27. doi: 10.1186/s13287-020-1551-z (PMC7055148; doi:10.1186/s13287-020-1551-z)
Supplement: Supplementary file 6 — Additional file 6 : Table S1. Fertility and mortality in HDC-/-mice the overall birth and mortality rates of pregnant WT and HDC-/- mice post pyrilamine intragastric administration during E8.5 to E18.5. [file 13287_2020_1551_MOESM6_ESM.docx]

|  | Total litters | Average litter size | The number of deaths |
| --- | --- | --- | --- |
| Con  H1R antagonist | 29  53 | 7.25  7.5 | 2  7 |

**Supplementary Table 1. Fertility and mortality in HDC^-/-^mice**

The overall birth and mortality rates of pregnant WT and HDC^-/-^ mice post pyrilamine intragastric administration during E8.5 to E18.5.
