# Supplementary material for: Disruption of histamine/H1R signaling pathway represses cardiac differentiation and maturation of human induced pluripotent stem cells
Source: Stem Cell Res Ther. 2020 Mar 4;11:27. doi: 10.1186/s13287-020-1551-z (PMC7055148; doi:10.1186/s13287-020-1551-z)
Supplement: Supplementary file 7 — Additional file 7 : Table S2. Quantitative real time PCR primers. [file 13287_2020_1551_MOESM7_ESM.docx]

**Supplementary Table 2. Quantitative real time PCR Primers**

| Primer | Forward | Reverse |
| --- | --- | --- |
| nkx2-5 | GCCGCCAACAACAACTTC | TACCAGGCTCGGATACCAT |
| TNNT2 | AAGAAGAAGATTCTGGCTGAGAG | ACTTTCTGGTTATCGTTGATCCT |
| MYH6 | TCAGCTGGAGGCCAAAAGTAAAGGA | TTCTTGAGCTCTGAGCACTCGTCT |
| MTH7 | TCGTGCCTGATGACAAACAGGAGT | ATACTCGGTCTCGGCAGTGACTTT |
| HRH1 | AGATGTGTGAGGGCAACAAGA | CAAGCAGATAGTGCTCAGGAC |
| HRH2 | CGTGTCCTTGGCTATCACTGA | GGCTGGTGTAGATATTGCAGAAG |
| MESP1 | AGAGCCTGACCAAGATCGAGACG | GCGTCCGTGTCTGCATCTGC |
| Brachyury | ACCCAGTTCATAGCGGTGAC | CCATTGGGAGTACCCAGGTT |
| GAPDH | GGAGCGAGATCCCTCCAAAAT | GGCTGTTGTCATACTTCTCATGG |
| CALM1 | GGGTCAGAACCCAACAGAAGC | AGACTCGGAATGCCTCACGG |
| CALM2 | GAGAGCGAGCTGAGTGGTTGTG | TTCAGTCAGTTGGTCAGCCTTGC |
| CALM3 | TCCGAGAGGCGTTCCGTGTC | ATCCACCTCCTCATCGGTCAGC |
| CASQ2 | GGCAGAAGAGGGGCTTAATTT | GAAGACACCGGCTCATGGTAG |
| RYR2 | GGCAGCCCAAGGGTATCTC | ACACAGCGCCACCTTCATAAT |
